# The complex ecology of genitalia: Gonopodium length and allometry in the Trinidadian guppy

**DOI:** 10.1002/ece3.7351

**Published:** 2021-03-18

**Authors:** José Jonathas Pereira Rodrigues de Lira, Yue Yan, Sophie Levasseur, Clint D. Kelly, Andrew P. Hendry

**Affiliations:** ^1^ Department of Biology and Redpath Museum McGill University Montreal QC Canada; ^2^ Faculty of Arts and Sciences Concordia University Montreal QC Canada; ^3^ Pavillon des Sciences Biologiques Université du Québec à Montréal Montréal QC Canada

**Keywords:** genital evolution, genitalia allometry, *Poecilia reticulata*, predation, static allometry

## Abstract

Male genitalia present an extraordinary pattern of rapid divergence in animals with internal fertilization, which is usually attributed to sexual selection. However, the effect of ecological factors on genitalia divergence could also be important, especially so in animals with nonretractable genitalia because of their stronger interaction with the surrounding environment in comparison with animals with retractable genitalia. Here, we examine the potential of a pervasive ecological factor (predation) to influence the length and allometry of the male genitalia in guppies. We sampled guppies from pairs of low‐predation (LP) and high‐predation (HP) populations in seven rivers in Trinidad, and measured their body and gonopodium length. A key finding was that HP adult males do not have consistently longer gonopodia than do LP adult males, as had been described in previous work. However, we did find such divergence for juvenile males: HP juveniles have longer gonopodia than do LP juveniles. We therefore suggest that an evolutionary trend toward the development of longer gonopodia in HP males (as seen in the juveniles) is erased after maturity owing to the higher mortality of mature males with longer gonopodia. Beyond these generalities, gonopodium length and gonopodium allometry were remarkably variable among populations even within a predation regime, thus indicating strong context dependence to their development/evolution. Our findings highlight the complex dynamics of genitalia evolution in Trinidadian guppies.

## Graphical abstract

Divergent ecological selection drives populations to evolve different trait values. Here, we evaluated whether the length and the allometry of the male guppy genitalia diverged between low‐ and high‐predation habitats. We found that the male genitalia is consistently longer only among high predation juveniles, but among adults, results were quite variable, such as for genitalia allometry.

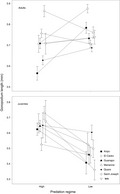

## 1 INTRODUCTION

Male genitalia in animals, especially those with internal fertilization, exhibit a remarkable pattern of rapid divergence (Arnqvist, 1998; Hosken et al., 2019; Hosken & Stockley, 2004; Simmons, 2014). This phenomenon has been overwhelmingly attributed to sexual selection (Arnqvist, 1998; Eberhard et al., 2009, 2018; Hosken & Stockley, 2004; Simmons, 2014), whereas the role played by other factors, such as ecological variation, is less often studied (Langerhans et al., 2016). One reason for this bias in effort might be the typical assumption that male genitalia do not interact much with the surrounding environment—and therefore would not be expected to show much of an ecological signature. That is, male genitalia tend to be small or are usually hidden away (for a review, see Kelly & Moore, 2016), such as when not engorged with blood (e.g., humans—Yuh & Shindel, 2017), when retracted inside the body (e.g., some mammals and crocodilians—Kelly & Moore, 2016), or when not hydrostatically inflated (e.g., some turtles, crocodilians, birds, and mammals—Kelly, 2007). Then, typically just before or during copulation, part of the genitalia can be rapidly enlarged to facilitate sperm transfer inside females. Postcopulation, the genitalia are then often retracted or deflated and hidden away once more (Kelly & Moore, 2016). Under these conditions, one might expect ecologically based selection on male genitalia to be relatively modest, restricted to indirect costs such as variation in energy limitation or risk of infection. By contrast, frequently studied ecological drivers that tend to impose direct selection on traits, such as temperature or moisture or predation or intraspecific competition, would seem likely to be of relatively little importance to the evolution of male genitalia.

To make rapid initial progress on understanding the potential ecological drivers of male genital evolution, we therefore need to start with a special system. *Poecillid* fishes represent such a system because male genitalia cannot be deflated or hidden inside the body, but rather only moved to a different (but still external) position. Specifically, the gonopodium of *Poecillid* fishes is used to transfer sperm to the female during copulation (Houde, 1997; Magurran, 2005), in which case it moves from a resting position to a copulatory position so that the tip of the gonopodium is inserted in the female genital pore (Rosen & Tucker, 1961). After copulation, the gonopodium is then moved back to a resting position along the underside of the body. Although this continual exposure of male genitalia even outside of copula is an exception to the general rule for animals, it still characterizes more than 250 species in the evolutionarily diverse *Poecillid* radiation (Parenti, 1981; Stockwell & Henkanaththegedara, 2011). Further, insights from this system could motivate work on species where male genitalia remain continuously exposed at something less than their full size, such as in some mammals (Kelly & Moore, 2016). Under such conditions, we might reasonably expect male genitalia to “have an ecology” shaping among‐population variation in response to spatial variation in putative selective forces. Finally, studies of genital ecology in these groups will form an important point of comparison for assessing the drivers of genetic evolution in species with usually more cryptic ecology.

An important selective force shaping the ecology of numerous traits in numerous organisms is predation—both its intensity (e.g., rate of mortality) and type (e.g., aerial versus aquatic, pursuit versus ambush, and the specific predator species). In the case of continuously exposed male genitalia, such as in *Poecillid* fishes, predation could have direct or indirect selective effects. As an example of a direct effect, shorter genitalia might evolve under high‐predation risk so that escape ability is not compromised by long genitalia. For instance, in the poecilid species *Gambusia affinis* (Baird & Girard, 1853), males with a longer gonopodium had a slower burst‐swimming speed, suggesting increased susceptibility to predation (Langerhans et al., 2005). As an example of an indirect effect, longer genitalia might evolve under high‐predation risk to increase the success of rapid “sneaky” matings—as opposed to courtship that might increase predation risk. Indeed, poecilid species that employ sneaky copulations only have longer gonopodium than species that use courtship as the primary mating tactic (Jennions & Kelly, 2002; Rosen & Tucker, 1961). Of course, selection shapes many other aspects of genitalia, such as shape (Arnqvist, 1998; Simmons, 2014). Regardless, the study of the gonopodium length in *Poecillid* fishes presents a useful system for studying how male genital evolution can be shaped by ecological variation among populations (Broder et al., 2020; Langerhans et al., 2005).

If a given body part diverges in relative size among populations, that divergence must be accomplished by changes in rates or patterns of relative growth of that specific part. Thus, like variation in the relative size of any other body part, the ecology of genitalia should be reflected in the evolution of allometry, that is, changes in the rate of increase in trait size relative to increasing body size (Bonduriansky, 2007; Eberhard, 2009). Hence, if ecological differences among populations favor different relative gonopodium length, we would expect corresponding differences in allometric coefficients. For instance, the above‐described expectation of shorter gonopodia in higher predation environments (Langerhans et al., 2005) should lead to the evolution of shallower gonopodium allometry relative to guppies in lower predation environments. On the other hand, the above‐described alternative expectation of longer genitalia in higher predation environments (Kelly et al., 2000) should lead to the evolution of steeper allometries.

### 1.1 Trinidadian guppies

Guppies are a promiscuous live‐bearing fish in which male genitalia are a modified anal fin known as the gonopodium (Houde, 1997; Magurran, 2005). Male guppies possess two alternative mating tactics: They can either court and copulate with a receptive female or they can attempt a sneaky copulation, in which case a male approaches a female from behind and thrusts its gonopodium into the female urogenital pore without obvious consent (Godin, 1995; Houde, 1997; Kelly et al., 2000; Magurran, 2005). In the former case, females choose mates based on multiple morphological, behavioral, and social aspects (Houde, 1997), whereas evidence for female preference based on the gonopodium is contradictory in guppies (Brooks & Caithness, 1995; Gasparini et al., 2011). Among other poecilids, males actively display the gonopodium to females (Basolo, 1995; Langerhans et al., 2005), and strong empirical evidence suggests that *Gambusia* females prefer males with longer gonopodia (Kahn et al., 2010; Langerhans et al., 2005)—although this result is not apparent when only small males are considered (Kahn et al., 2010).

For the ecological context of our study, guppies inhabit low‐predation (LP) or high‐predation (HP) habitats in the Northern Range of Mountains in Trinidad. These habitats are classified as LP versus HP based on the absence versus presence of piscivorous fishes (Endler, 1980; Reznick et al., 1996). Many studies have validated the utility of this LP versus HP contrast, including multiple demonstrations of higher mortality rates in HP environments than in LP environments (Gordon et al., 2009; Reznick et al., 1996; Weese et al., 2011). Moreover, guppies are known to diverge in manifold morphological, behavioral, and life‐history traits between populations inhabiting these two environment types in multiple rivers in Trinidad (Endler & Houde, 1995; Godin, 1995; Houde, 1997; Magurran, 2005). Of most relevance to our research questions, guppies occupying HP habitats mature earlier and at smaller sizes (Magurran, 2005; Reznick & Endler, 1982) and possess longer gonopodia than do their LP counterparts (Kelly et al., 2000).

### 1.2 Predictions

An antecedent to our study was the work of Kelly et al. (2000), which reported that adult HP males have longer gonopodia than do adult LP males. We therefore first predicted a similar pattern for adult males in our more comprehensive paired‐population analysis (see study design below). Correspondingly, then, we next predicted that the allometric growth of the gonopodium would be steeper in HP males than in LP males (Magurran, 2005; Reznick & Endler, 1982). To help explain the patterns observed, we further considered how results varied through ontogeny (i.e., gonopodium length and allometry in juveniles versus adults) and in relation to age at maturity of different populations, since it is a factor known to influence the relative growth of the male genitalia, such as demonstrated in crabs (Lira et al., 2015). Note, however, that we do not investigate the specific selective causes of predation‐associated divergence, such as changes in maneuverability or visibility or correlated consequences of changes in mating behavior. Discriminating among these and other specific mechanisms will require focused experimental work informed by the overall patterns we here demonstrate.

## 2 MATERIALS AND METHODS

### 2.1 Sampling and fish care

We sampled juvenile and adult male guppies in one low‐ and one high‐predation locality in each of seven different streams in the Northern Mountain Range, Trinidad. These localities were classified as low‐ and high‐predation localities based on the absence or presence of piscivorous fish, respectively (Endler, 1978; Gotanda & Hendry, 2014; Kelly et al., 2000; Reznick & Endler, 1982). All fish were transported to our laboratory at the William Beebe Tropical Research Station in Trinidad, acclimatized for 30 min, transferred to 20 L aquariums, and immediately treated for bacterial, fungal, and parasitic infections with Polyguard™ (Seachem Laboratories, Inc.). Fish were fed live brine shrimp or flake food if they remained more than 24 hr in the laboratory, but most fish were released back to their original site the day after processing (details of processing below). All fish were kept at 20–24°C and on a natural 12:12 (light:dark) photoperiod. All fish handling was in accordance with McGill Animal Use Protocol No. 4570.

### 2.2 Measurements and maturity status

Body length (from snout to caudal peduncle) and gonopodium length (from base of gonopodium to distal tip, excluding the hood—see Kelly et al., 2000) were obtained from digital photographs using the software ImageJ (Abràmoff et al., 2004). We first anesthetized the fish with an aqueous solution of tricaine methanesulfonate (MS‐222) and NaHCO_3_ and then placed them on their right side on a white background containing a ruler. We then photographed the left side of each fish with a Nikon D300 Digital Camera equipped with a 60 mm macro lens, with illumination provided by two full‐spectrum fluorescent lights and a Nikon Speedlight Commander Kit R1C1 Flash.

**FIGURE 1:**
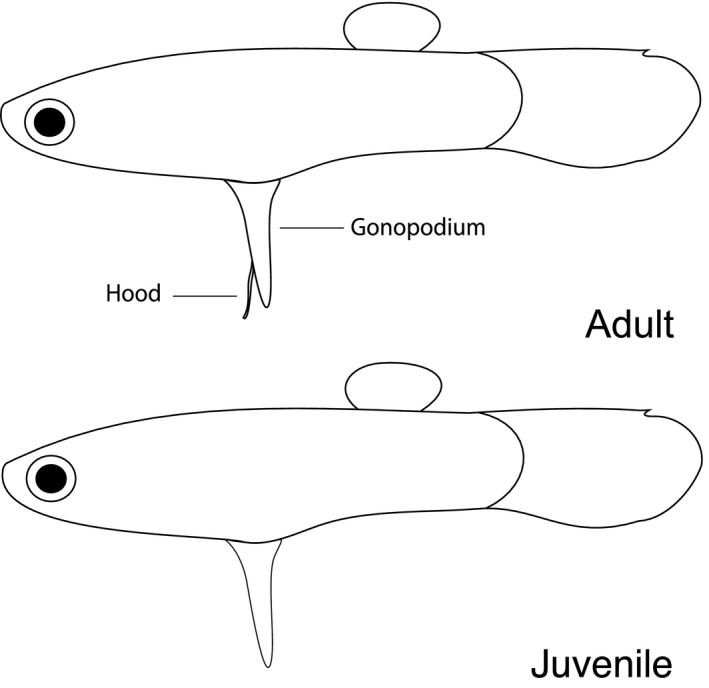
Scheme of the stages of gonopodium development of *Poecilia reticulata*, demonstrating the final stage (adults) and the advanced stage of development (juveniles)

The development stage of males was determined based on the stage of development of the hood, a sensory protuberance in the gonopodium (Houde, 1997), which was visualized under a Leica ES2 stereomicroscope before the photographs were taken. Males were categorized as mature when the hood extended beyond the distal tip of the gonopodium (the hook), and immature when the hood was shorter than the gonopodium (Houde, 1997). Furthermore, we visually classified the development of the gonopodium into three different stages (Figure 1): (a) early stage, when the differentiation of the anal fin into the gonopodium is ongoing and it bears a wide base, forming a triangular shape—not shown in Figure 1; (b) advanced stage, including the substages “*Hood not developed,*” when the gonopodium has developed a thinner base—what remains henceforth—and has acquired an appearance of a fully developed gonopodium, similar to the “*Hood developing*” substage and the “*Final stage*” of development, as described below; and “*Hood developing,*” representing the phase in which the hood has just started to develop until the phase in which it has acquired a filament‐like shape but is still shorter than the gonopodium; and finally, (c) final stage, when the hood is fully developed and longer than the hook—typical from adult males. Although we initially distinguished the substages “*Hood not developed*” from “*Hood developing,*” we subsequently grouped them together as “advanced stage” because there was no apparent difference in the allometric growth between these stages. We removed early‐stage juveniles from the statistical analysis due to small sample sizes; therefore, we refer to advanced‐stage juveniles simply as juveniles henceforth.

### 2.3 Statistical analysis

All analyses were performed in the R statistical environment (R Core team, 2019) with a significance level of 5%. Following previous work (e.g., Kelly et al. 2000), our predictions about how the gonopodium length varies among populations were tested using an ANCOVA (type III sums of squares) implemented with log10‐transformed data in the package *emmeans* (Lenth, 2019). Specifically, gonopodium length was modeled (separately for adults and juveniles) as a function of body length (continuous explanatory variable), river (categorical fixed effect), predation (categorical fixed effect), and all possible interactions. The main effect of predation was used for testing differences between predation regimes (low or high predation) in average gonopodium length (standardized for body length). The main effect of river was used for inferences about river‐specific effects that were independent of predation (standardized for body length). The predation‐by‐river interaction was used for inferring context dependence (i.e., effect of river) in how predation influenced gonopodium length (standardized for body length). The body length‐by‐predation interaction was used for testing differences between predation regimes in gonopodium allometry—independent of river. The body length‐by‐river interaction was used to test for differences among rivers in allometry—independent of predation. Finally, the three‐way interaction was used to test for context dependence (i.e., effect of river) of predation influencing gonopodium allometry. We also implemented an ANCOVA (type I sum of squares) to calculate the least square means of gonopodium length, also using the package *emmeans* (Lenth, 2019). This model was identical to the ANCOVA (type III sum of squares) described above, but using raw data, rather than log10‐transformed data. Finally, we examined whether inferences from the above model held when enforcing homogenous slopes of the covariate (i.e., no interaction between body length and other factors in the model).

The above models are most appropriate for answering the questions raised in the introduction about effects of predation on gonopodium length and allometry. However, the best estimates of the precise value for allometry need to come from population‐specific analyses that allow for error in both the predictor (body length) and response (gonopodium length). We generated these best estimates of the allometric relationship between log10 body length and log10 gonopodium length (separately for adults and juveniles) for each population (i.e., each combination of river and predation) by implementing reduced major axis regressions (Standardized Major Axis) using the package *smatr* (Warton et al., 2012). We used log‐transformed data (body length and gonopodium length) because allometry is often described based on the allometric slope (*b*) of log–log regressions (log Y = log *a* + *b*log X) of the allometric equation Y = *a*X*^b^* (Bonduriansky & Day, 2003; Rodríguez et al., 2015). Isometry (*b* = 1) indicates that the trait grows in the same proportion as the body, negative allometry (*b* < 1) indicates that the trait grows proportionally slower than the body (i.e. larger individuals have relatively smaller traits), and positive allometry (*b* > 1) indicates that the trait grows proportionally faster than the body (i.e., larger individuals have relatively larger traits—(Bonduriansky & Day, 2003; Rodríguez et al., 2015).

Allometry estimates from these reduced major axis regressions were used for some data visualizations (as noted in the relevant figure captions) and for some further explorations of the contributors to gonopodium length and allometry—particularly size at maturation. Specifically, we estimated the size at sexual maturity for each population by calculating the size in which at least 50% of the males were classified as adults (L_50%_) using the package “*sizeMat*” (Torrejon‐Magallanes, 2019). We then tested whether the allometric slope (from reduced major axis regression) is influenced by the size at sexual maturity using log10‐transformed values in simple linear regression with size at sexual maturity as the explanatory variable and the allometric slope as the response variable, for both adults and juveniles.

## 3 RESULTS

**FIGURE 2:**
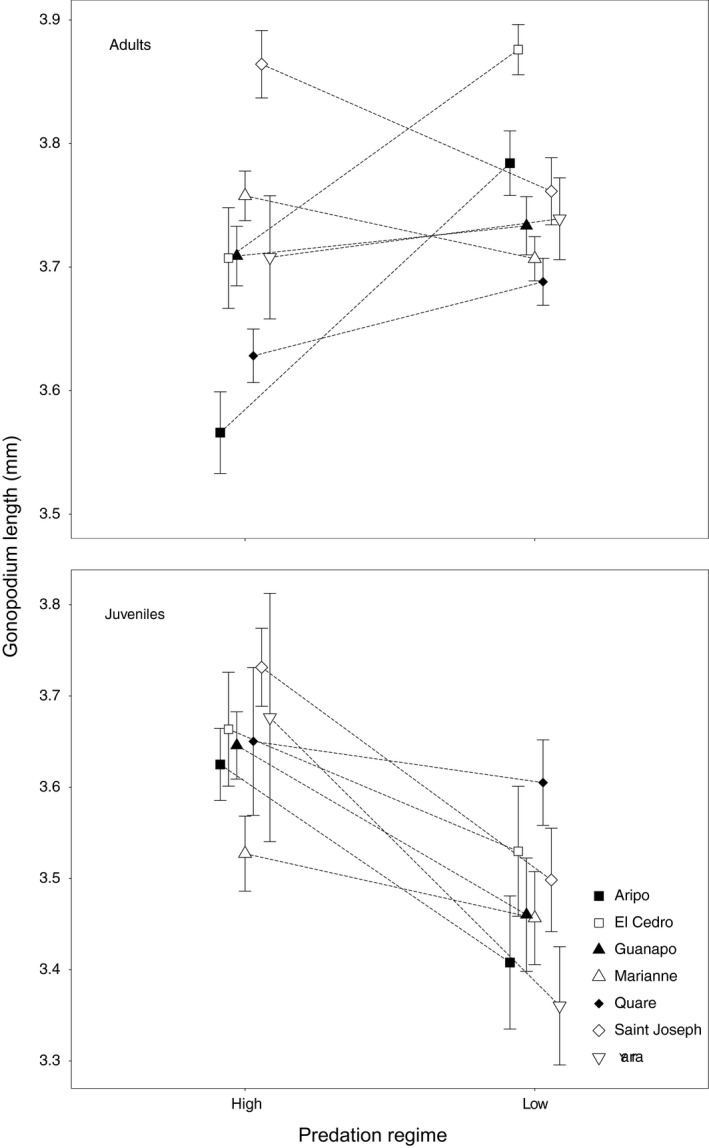
Least square means (±*SE*) of gonopodium length (mm) for adults and juveniles of *Poecilia reticulata*. These estimates were obtained from an ANCOVA (type I sum of squares) using the raw data of gonopodium length and body length

Contrary to Kelly et al. (2000), we found that relative gonopodium length (i.e., standardized for body length, henceforth “gonopodium length”) was not longer in HP adult males than in LP adult males. Instead, results bordered on the opposite outcome (*p* = .053); that is, gonopodium length was—if anything—shorter (on average) in HP adult males than in LP adult males (HP males: 3.69 ± 0.012 mm, LP males: 3.75 ± 0.009 mm; Table 1). The lack of significance here was most likely due to a strong predation‐by‐river interaction—signifying context dependence, that is, differences among rivers in gonopodium length of our sampled HP versus LP populations (Figure 2). By contrast, HP juveniles had (on average) longer gonopodia (standardized for body length) than LP juveniles (HP males: 3.67 ± 0.028 mm, LP males: 3.48 ± 0.025 mm; Table 1): this time with no context dependence (predation‐by‐river interaction). When removing interactions with body length from the model, thus enforcing homogeneous slopes among populations, the only change was that gonopodium length in juveniles now showed context dependence, that is, a predation‐by‐river interaction (Table S1).

**TABLE 1:** Results of a two‐way analysis of variance evaluating the influence of log10 body length, predation regime, river, and their interaction on log10 gonopodium length in *Poecilia reticulata*

	*F*	*df*	*p*‐value
Adults			
log (body length)	**307.7**	**1**	**<.001**
Predation	3.74	1	.053
River	**7.58**	**6**	**<.001**
log (body length) * Predation	**10.45**	**1**	**.0013**
log (body length) * River	1.84	6	.088
Predation * River	**6.69**	**6**	**<.001**
log (body length) * Predation * River	**2.42**	**6**	**.025**
Residuals		1,157	
Juveniles			
log (body length)	**180.8**	**1**	**<.001**
Predation	**16.41**	**1**	**<.001**
River	1.14	6	.33
log (body length) * Predation	0.1	1	.75
log (body length) * River	1.44	6	.19
Predation * River	0.94	6	.46
log (body length) * Predation * River	**3.43**	**6**	**.002**
Residuals		705	

Note

Bold indicates significant *p*‐value.

For allometry, we found a main effect of body length and a three‐way interaction among body length, predation, and river for both juveniles and adults (Table 1; Figures S1 and S2). For adults, we further found a two‐way interaction between body length and predation (Table 1; Figure S1). The lack of an overall effect of predation is likely due to the fact that differences between HP and LP populations within rivers were not consistent across rivers (Table 2; Figures S1 and S2). This context dependence was evident between predation regimes (two‐way interaction between body length and predation) for adults and in the three‐way interaction for both juveniles and adults.

**TABLE 2:** Reduced major axis regression analysis between body length (explanatory variable) and gonopodium length (response variable) of *Poecilia reticulata* sampled in low‐predation and high‐predation habitats in seven rivers in Trinidad

River	Stage	Predation	*n*	Intercept (±CI)	Slope (±CI)	*R* ^2^	*p*
Aripo	Adults	LP	101	−0.60 (−0.83, −0.37)	0.96 (0.79, 1.17)	0.04	<.001
		HP	126	−0.51 (−0.68, −0.34)	0.91 (0.77, 1.06)	0.19	<.001
	Juveniles	LP	31	−2.55 (−3.69, −1.41)	2.58 (1.80, 3.69)	0.07	.15
		HP	122	−1.51 (−1.85, −1.17)	1.80 (1.53, 2.13)	0.16	<.001
El Cedro	Adults	LP	90	−0.24 (−0.41, −0.06)	0.68 (0.55, 0.84)	0.01	.39
		HP	59	−0.69 (−0.98, −0.4)	1.06 (0.84, 1.34)	0.22	<.001
	Juveniles	LP	63	−2.13 (−2.64, −1.61)	2.28 (1.89, 2.75)	0.45	<.001
		HP	40	−2.28 (−3.11, −1.45)	2.47 (1.85, 3.29)	0.20	.003
Guanapo	Adults	LP	122	−0.36 (−0.51, −0.2)	0.76 (0.65, 0.89)	0.21	<.001
		HP	58	−0.24 (−0.43, −0.05)	0.68 (0.54, 0.86)	0.23	<.001
	Juveniles	LP	55	−1.54 (−2.02, −1.06)	1.77 (1.41, 2.21)	0.33	<.001
		HP	83	−1.50 (−1.93, −1.08)	1.78 (1.45, 2.2)	0.13	<.001
Marianne	Adults	LP	106	−0.23 (−0.37, −0.1)	0.67 (0.56, 0.79)	0.26	<.001
		HP	88	−0.34 (−0.48, −0.2)	0.77 (0.66, 0.89)	0.50	<.001
	Juveniles	LP	41	−2.75 (−3.51, −2.0)	2.83 (2.26, 3.56)	0.49	<.001
		HP	51	−2.27 (−3.04, −1.49)	2.41 (1.84, 3.16)	0.08	<.001
Quare	Adults	LP	94	−0.32 (−0.46, −0.18)	0.74 (0.63, 0.87)	0.44	<.001
		HP	80	−0.32 (−0.48, −0.16)	0.74 (0.62, 0.89)	0.32	<.001
	Juveniles	LP	43	−1.88 (−2.61, −1.15)	2.10 (1.56, 2.83)	0.09	.047
		HP	26	−2.84 (−4.03, −1.66)	2.97 (2.1, 4.18)	0.30	.003
Saint Joseph	Adults	LP	75	−0.37 (−0.56, −0.18)	0.78 (0.64, 0.95)	0.27	<.001
		HP	55	−0.33 (−0.54, −0.13)	0.77 (0.62, 0.97)	0.31	<.001
	Juveniles	LP	48	−1.59 (−2.17, −1.02)	1.81 (1.39, 2.36)	0.19	.002
		HP	67	−1.80 (−2.23, −1.37)	2.05 (1.71, 2.45)	0.46	<.001
Yarra	Adults	LP	65	−0.42 (−0.65, −0.19)	0.82 (0.65, 1.02)	0.18	<.001
		HP	66	−0.70 (−0.97, −0.43)	1.08 (0.87, 1.34)	0.26	<.001
	Juveniles	LP	26	−2.29 (−3.43, −1.14)	2.39 (1.61, 3.54)	0.08	.15
		HP	37	−2.39 (−3.21, −1.57)	2.61 (1.97, 3.45)	0.32	<.001

Note

These stage‐ and population‐specific regressions are intended to generate the best possible estimates of allometry in each case. The testing of predictions, by contrast, relies on the results reported in Table 1.

As expected, we found that LP males are larger (body length) than HP males, both in adults (LP males: 16.5 ± 0.045, HP males: 14.98 ± 0.051 mm; ANCOVA: *F*_1,1,171_ = 496.28, *p* < .001) and juveniles (LP males: 15.36 ± 0.054, HP males: 14.01 ± 0.049 mm; ANCOVA: *F*_1,719_ = 337.69, *p* < .001). We also found a main effect of river (adults: *F*_1,1,171_ = 16.24, *p* < .001; juveniles: *F*_1,719_ = 14.92, *p* < .001) and a predation‐by‐river interaction (adults: *F*_1,1,171_ = 31.03, *p* < .001; juveniles: *F*_1,719_ = 25.8, *p* < .001).

Population‐specific reduced major axis regression estimates of allometry varied considerably among populations and between juveniles and adults (Table 2). Overall, gonopodium allometry for adults was negatively allometric (slope < 1), indicating that larger adult males have shorter gonopodia relative to their body size than do smaller adult males. By contrast, the pattern of allometry among juveniles was positive (slope > 1; Table 2), indicating that larger juvenile males have longer gonopodia relative to their body size than do than smaller juvenile males.

**FIGURE 3:**
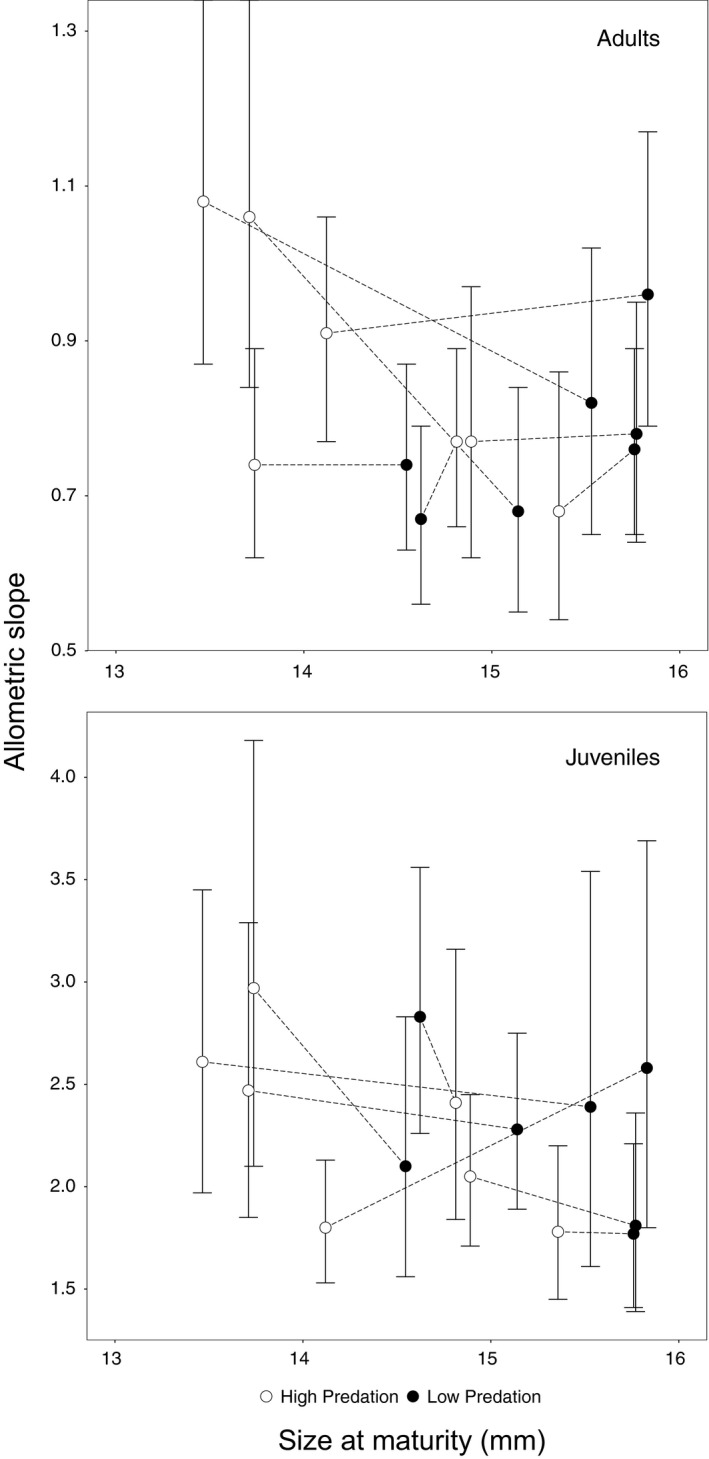
Relationship between size at sexual maturity and reduced major axis regression allometric slopes (±*SD*) of the gonopodium relative to body length for adults and juveniles of *Poecilia reticulata*. Size at maturity was estimated based on the size in which at least 50% of the sampled males from each population were classified as adults (L_50%_)

As expected, we found that HP males generally (but not universally) mature at a smaller body length than do LP males (Figure 3). Contrary to our prediction, however, we did not find a relationship between the size at sexual maturity and the allometric slopes for juveniles or adults (Figure 3); that is, populations with a smaller size at maturity did not have steeper slopes, nor did populations with a larger size at maturity have lower slopes (juveniles: slope = −1.47, *R* ^2^ = 0.21, *p* = .1; adults: slope = −1.2, *R* ^2^ = 0.18, *p* = .13; Figure 3). We did find, however, a negative relationship for adults among HP populations (slope = −2.81, *R* ^2^ = 0.6, *p* = .041), partially supporting our prediction, but no trend was observed for HP juveniles (slope = −2.6, *R* ^2^ = 0.45, *p* = .1). We also did not find a relationship between size at sexual maturity and the allometric slopes within LP populations in juveniles (slope = −1.92, *R* ^2^ = 0.16, *p* = .38) or in adults (slope = 2.3, *R* ^2^ = 0.46, *p* = .09).

## 4 DISCUSSION

We explored a potential ecological factor—predation—shaping gonopodium evolution by examining the variation in gonopodium length within and among populations of Trinidadian guppies. Earlier work (Kelly et al., 2000) had reported that adult HP males have consistently longer gonopodia (standardized for body length) than do adult LP males. We found that this difference pointed in the opposite direction, that is, HP adult males have shorter gonopodium than LP adult males—on average, although the variation among populations within a predation regime was high. Not surprisingly then (in light of these new results), we also found that gonopodium allometry was not consistently steeper in HP populations than in LP populations (Table 2). Hence, we explored other possible drivers of among‐population variation—most obviously size at maturity—yet this trait also did not explain variation in gonopodium length or allometry (Figure 3).

Examination of stage‐specific average gonopodium length provides new insights that suggest a possible resolution to the above set of diverse observations. In particular, juvenile HP males consistently had longer gonopodia (for a given body length) than did juvenile LP males within rivers, indicating that the classic HP versus LP distinction from Kelly et al. (2000) was present before maturity but disappeared afterward. Based on these findings, we first suggest that the reason HP allometry is not steeper than LP allometry is simply that the differences in body length start to arise very early during development. We next suggest that juvenile gonopodium length does indeed reflect the true evolutionary expectations—gonopodia are developmentally larger in HP males than in LP males—but that environmental effects erase this association after sexual maturity. In particular, we suggest that males with longer gonopodia have higher mortality rates in HP habitats, thus environmentally degrading the evolutionary difference in gonopodium length between LP and HP populations. In the following sections, we further explain these ideas and some alternatives, and we address other interesting discrepancies and patterns.

### 4.1 Why do our results differ from previous work?

Based on Kelly et al. (2000), we expected to find that HP adult males have longer gonopodia than do LP adult males. Instead, our paired (by river) HP‐LP design showed highly river‐specific outcomes, wherein HP adult males had longer gonopodia than LP adult males in one river (Saint Joseph), LP adult males had longer gonopodia than HP adult males in three rivers (Aripo, El Cedro, and Yarra), and no difference was evident in three other rivers (Guanapo, Marianne, and Quare; Figure 2). Further examination of the specific populations studied in Kelly et al. (2000) versus our current work revealed that the different results between studies reflect our more extensive sampling and our explicitly replicated and paired HP‐LP design.

In particular, the finding of Kelly et al. (2000) that HP males have longer gonopodia than do LP males was heavily shaped by two HP populations (Guanapo and Tacarigua) that have exceptionally long gonopodia. By contrast, the other HP populations examined in their work had gonopodium lengths that were similar to, or in one river even shorter than, some LP populations (Figure 2 in Kelly et al., 2000). In short, the lack of a paired design (i.e., LP and HP populations sampled in each of multiple rivers) made their study unable to disentangle the effect of river from the effect of predation. Thus, reconciliation between this previous work and our current study lies in the fact that adult males from some rivers have longer gonopodia than do those from other rivers, regardless of predation regime; and Kelly et al. (2000) happened to sample HP populations from two of the rivers where males have very long gonopodia. However, as we will explain below, examination of juvenile gonopodia will recover findings consistent with the original hypothesis and conclusion by Kelly et al. (2000), which therefore motivates additional hypotheses worthy of future study.

### 4.2 Reconciling diverse outcomes with a new hypothesis

Our results initially might seem a contradictory mix of outcomes: HP juveniles have longer gonopodia than do LP juveniles, HP adults do not have longer gonopodia than do LP adults, and allometries overall do not differ between the two predation regimes in adults nor in juveniles. Moreover, quantitative variation among populations in size at maturity does not explain gonopodium length or allometries—and therefore cannot explain the patterns of HP‐LP divergence. Further consideration has led us to a new hypothesis that could reconcile these observations in an interesting way.

We suggest that HP males are indeed favored by selection to have longer gonopodia and that they achieve this outcome by having longer gonopodia throughout development up to sexual maturity. These differences arise so early in development, or diverge so gradually, that they do not generate statistically detectable differences in allometry. We next suggest that, once sexually mature, males with longer gonopodia experience higher mortality rates—especially in HP habitats. This higher mortality of males with longer gonopodia could be expected (a) due to reduced swimming ability associated with longer gonopodia—as seen for mosquitofish (Langerhans et al., 2005) or (b) because males with longer gonopodia engage more frequently in courtship (Reynolds et al., 1993; but see Kwan et al., 2016), which should be a riskier behavior in HP habitats. Under this hypothesis, the ecologically driven evolutionary difference in gonopodium growth is erased by differential mortality following maturity.

This new hypothesis represents a form of counter‐gradient variation, where within‐generation environmental and between‐generation evolutionary effects act in opposite directions (Conover & Schultz, 1995; Grether et al., 2005). That is, evolution increases gonopodium length in HP populations for the reasons classically hypothesized (see Introduction), and as we have shown for juveniles, while a later‐acting (after maturity) environmental effect of differential predation eventually erases that evolutionary signature. Previous work has also invoked counter‐gradient effects for other guppy traits—specifically male color (Grether et al., 2005) and gene expression (Ghalambor et al., 2015). Our new hypothesis could be tested by examining gonopodium length through development for HP and LP populations in a common garden, where direct effects of predation are absent. It would also be valuable to conduct mark–recapture experiments in nature where the gonopodium length of individual males was measured and its effects on survival quantified in multiple HP and LP populations—as Weese et al. (2010) did for guppy color.

Finally, we thank the two reviewers of this paper for suggesting alternative hypotheses for the complex patterns we observed—hypotheses that relate to differential timing in the cessation of the growth of the gonopodium versus the body. For instance, our result could be obtained if (a) male guppies continue to grow after the gonopodium is fully developed, (b) this tendency is more pronounced in HP males than in LP males, and (c) gonopodium length does not change (much) after maturation. In such a scenario, (a) allometries might not differ much between predation regimes, (b) LP juveniles might have longer gonopodia than HP juveniles, but (c) this pattern might disappear or reverse in adults. Although we cannot conclusively eliminate this alternative explanation for the patterns we observed, it does not easily conform to known patterns of guppy growth. In particular, male guppies do not grow much (if at all) after maturity and the evidence does not suggest that any such growth is greater for HP than LP males (Handelsman et al., 2013; Reznick & Bryant, 2007). Further, Broder et al. (2020) demonstrated that guppies raised to adulthood with predator cues did not have longer gonopodia than guppies raised in the absence of predator cues.

### 4.3 Nonparallelism and context dependence

Regardless of the specific reason for differences in the average gonopodium length and allometry between HP and LP populations, it is important to emphasize the dramatic among‐population variation within each predation regime. That is, context dependence (i.e., river‐specific selective or environmental effects) appears to be strongly modifying phenotypic (and presumably evolutionary) outcomes away from deterministic parallelism in relation to predation. Such context dependence leading to substantial nonparallelism relative to predation is increasingly being reported for guppies (Fitzpatrick et al., 2015; Kemp et al., 2018), for other fishes (Oke et al., 2017; Stuart et al., 2017), and in general (Bolnick et al., 2018). Our results thus indicate another trait through which to consider the role of context dependence in causing deviations from deterministic parallel evolution in response to a particular dichotomous categorization, such as HP versus LP. We now discuss five potential contributors to such context dependence: (a) the selective pressure of predation is spatially and temporally variable, (b) predation is not the only important selective force, (c) habitat selection by guppies might alter the risk of predation, (d) gene flow between LP and HP populations within rivers might influence the extent of divergence, and (e) sexual selection might not strongly correlate with ecology.

First, spatiotemporal variation in predation intensity is well described for Trinidadian guppies, and it can have important implications for adaptive divergence (Endler, 1978, 1995; Millar et al., 2006). Such variation might have influenced our results in two primary ways. First, spatial variation in predation intensity is evident within and among rivers of a given predation regime because different predators are found in different rivers and at different locations within rivers (Endler, 1978; Magurran, 2005; Millar et al., 2006). Second, the population density of predators can vary seasonally (Magurran, 2005), likely due to variation in food availability and rainfall, resulting in temporal variation in predation risk. Hence, among‐population variation in gonopodium length within a predation regime might reflect variation in the type and intensity of predation—as has been argued for other guppy traits (Endler, 1978; Endler & Houde, 1995; Millar & Hendry, 2012; Millar et al., 2006).

Second, many ecological factors other than predation might be important, such as food availability. For instance, Schwab and Moczek (2016) demonstrated that nutrient limitation lead to smaller genitalia across different body sizes in two species of horned beetles; however, no effect of diet on genitalia development was detected in dung beetles (House & Simmons, 2007) or broad‐horned beetles (House et al., 2016). In guppies, recent empirical evidence does demonstrate that the development of the gonopodium is affected by food availability, with males raised under low food treatment developing longer gonopodium than males raised under high food treatment (Broder et al., 2020). Variation in food availability is common for guppies (Endler, 1995; Grether et al., 2001; Reznick et al., 2001), what can result in different levels of intraspecific competition, and consequently influence gonopodium development—as it does for color and life history in guppies (Grether et al., 2001; Reznick et al., 2001).

Third, guppies might actively select habitat patches or activity times in relation to immediate predation risk (Banet et al., 2016; Reynolds et al., 1993) or resource distribution—as seen in a variety of organisms (Gilliam & Fraser, 1988; Milinski, 1986). Such habitat selection might influence the effect of predation on traits as a whole, including the gonopodium. In fact, guppies are often seen along the riverbank in HP habitats (Reznick et al., 2001; Seghers, 1973), which should reduce the risk of predation since predators might not be able to swim in such shallow waters, while still being effective for resource acquisition. These site selection behaviors must surely also vary among sites of a given predation regime—as evidenced by river‐specific behavioral response to predation (Jacquin et al., 2016; Magurran, 2005).

Fourth, gene flow due to the downstream movement of LP guppies into HP habitats (Blondel et al., 2019; Crispo et al., 2006; Fitzpatrick et al., 2015) might hamper strong parallel divergence in gonopodia by increasing the frequency of LP‐origin males in HP habitats. As the rate of downstream movement is likely to vary among rivers (Blondel et al., 2019; Crispo et al., 2006; Fitzpatrick et al., 2015), and as our HP and LP sites were separated by different distances in different rivers, gene flow might well have influenced the direction and extent of the differences between our LP and HP populations. However, several recent studies have emphasized that the effects of gene flow do not seem to propagate far beyond immediate LP‐HP contact zones (Blondel et al., 2019; Fitzpatrick et al., 2015).

Fifth, some aspects of sexual selection might differ among rivers in ways that are not closely tied to predation—and this sexual selection might influence gonopodium evolution. For instance, a variety of studies have shown that male color varies dramatically among populations of a given predation regime (Endler & Houde, 1995; Gotanda & Hendry, 2014; Kemp et al., 2018; Millar & Hendry, 2012; Weese et al., 2010)—and the most logical explanation is different trajectories for the coevolution of male traits and female preferences—trajectories that are not closely linked to the classic HP versus LP contrast. The same population‐specific coevolution of sexually selected traits could quite reasonably be true for gonopodia since female guppies might be able to choose males also based on the gonopodium—although the empirical evidence is seemingly contradictory (Brooks & Caithness, 1995; Gasparini et al., 2011).

Additionally, across poecilid species, the length of the gonopodium is negatively related to the rate of courtship behavior (Furness et al., 2019; Rosen & Tucker, 1961); that is, species with longer gonopodia exhibit reduced courtship behavior. This pattern could have potentially influenced our findings, considering that rates of courtship behavior might diverge between LP and HP habitats—although the empirical evidence is contradictory (Farr, 1975; Magurran & Seghers, 1994; Houde, 1997—pag 91–94). However, although this is a well‐established trend across poecillid species, such a relationship does not seem to hold for within‐species comparisons (Ptacek & Travis, 1998). This phenomenon might be a useful area for future work given that the rate of courtship behavior likely varies among populations within a given predation regime (i.e., it is context‐dependent), for instance, due to spatial variation in the type and density of predators (Endler, 1978; Magurran, 2005; Millar et al., 2006).

## 5 CONCLUSION

Our work shows that the length and allometry of the gonopodium in guppies are highly variable within and among populations, even within a given predation regime. This variation appears to be driven by a diversity of effects. Through ontogeny, we suggest that different outcomes are the result of opposing short‐term environmental effects and longer‐term evolutionary effects—a form of counter‐gradient variation. Among populations, we suggest that different outcomes are driven not just by predation but also by other context‐specific outcomes, such as resource availability (see also Broder et al. 2020), variable types, and densities of predators, and presumably other environmental factors such as water clarity and flow rates. Within populations at a given stage of development, variation can also be highly, perhaps reflecting individual‐level genetic or environmental effects. Hence, we suggest that additional studies focusing on within‐ and among‐population variance in gonopodium length might prove an interesting substrate for exploring how complex ecologies interact with development to shapes patterns of trait variation.

## CONFLICT OF INTEREST

None declared.

## AUTHOR CONTRIBUTIONS

**José Jonathas Pereira Rodrigues de Lira:** Conceptualization (lead); data curation (lead); formal analysis (lead); investigation (lead); methodology (lead); project administration (lead); software (lead); validation (lead); writing–original draft (lead); writing–review and editing (lead). **Yue Yan:** Data curation (supporting); methodology (supporting); validation (supporting); writing–review and editing (supporting). **Sophie Levasseur:** Data curation (supporting); methodology (supporting); software (supporting); writing–review and editing (supporting). **Clint D. Kelly:** Formal analysis (supporting); investigation (supporting); supervision (supporting); writing–original draft (supporting); writing–review and editing (supporting). **Andrew P. Hendry:** Conceptualization (supporting); formal analysis (supporting); methodology (supporting); supervision (lead); visualization (supporting); writing–original draft (supporting); writing–review and editing (supporting).

## Supporting information

Appendix S1

Fig S1

Fig S2

## Data Availability

Data archived in DRYAD: https://doi.org/10.5061/dryad.b8gtht7bz.
