# Supplementary material for: The complex ecology of genitalia: Gonopodium length and allometry in the Trinidadian guppy
Source: Ecol Evol. 2021 Mar 18;11(9):4564–76. doi: 10.1002/ece3.7351 (PMC8093694; doi:10.1002/ece3.7351)
Supplement: Supplementary file 1 — Appendix S1 [file ECE3-11-4564-s001.docx]

Appendix

Figure S1 – Reduced major axis regression between log body length and log gonopodium length for high and low predation adults of *Poecilia reticulata* in each of seven rivers in Trinidad.

Figure S2 – Reduced major axis regression between log body length and log gonopodium length for high and low predation juveniles of *Poecilia reticulata* in each of seven rivers in Trinidad.

Table S1. Results of a two-way analysis of variance (type III sum of squares) evaluating the influence of body length, predation, river, and the interaction between river and predation on gonopodium length of adults in *Poecilia reticulata*.

|  | **F** | **d.f.** | **p - value** |
| --- | --- | --- | --- |
| **Adults** |  |  |  |
| log (body length) | 343.09 | 1 | < 0.001 |
| Predation | 18.01 | 1 | < 0.001 |
| River | 16.07 | 6 | < 0.001 |
| Predation * River | 12.98 | 6 | < 0.001 |
| Residuals |  | 1170 |  |
| **Juveniles** |  |  |  |
| log (body length) | 207.84 | 1 | < 0.001 |
| Predation | 19.16 | 1 | < 0.001 |
| River | 2.29 | 6 | 0.034 |
| Predation * River | 2.52 | 6 | 0.02 |
| Residuals |  | 718 |  |
