# Supplementary figures and images for: The complex ecology of genitalia: Gonopodium length and allometry in the Trinidadian guppy
Source: Ecol Evol. 2021 Mar 18;11(9):4564–76. doi: 10.1002/ece3.7351 (PMC8093694; doi:10.1002/ece3.7351)

log Gonopodium length (mm)

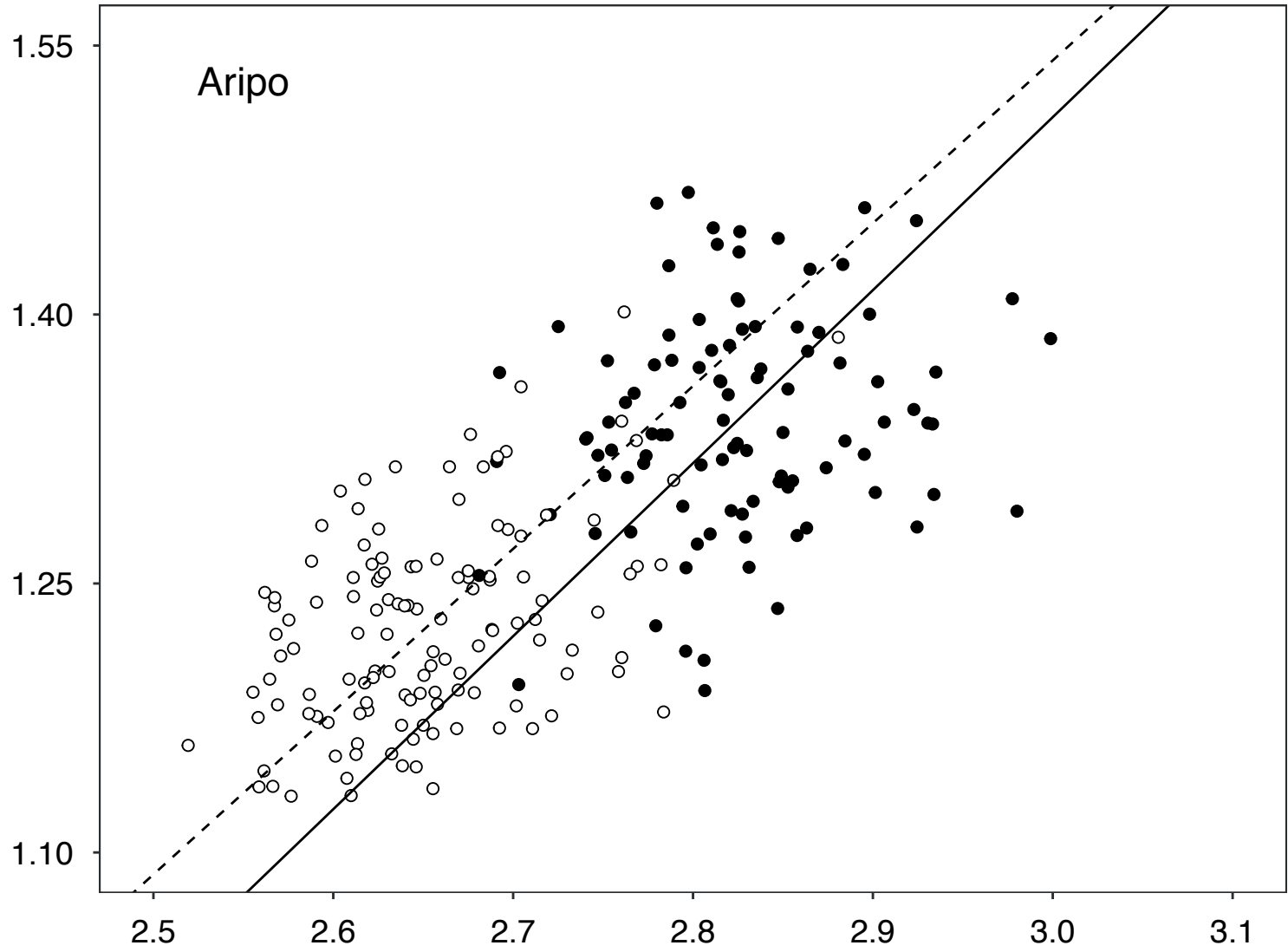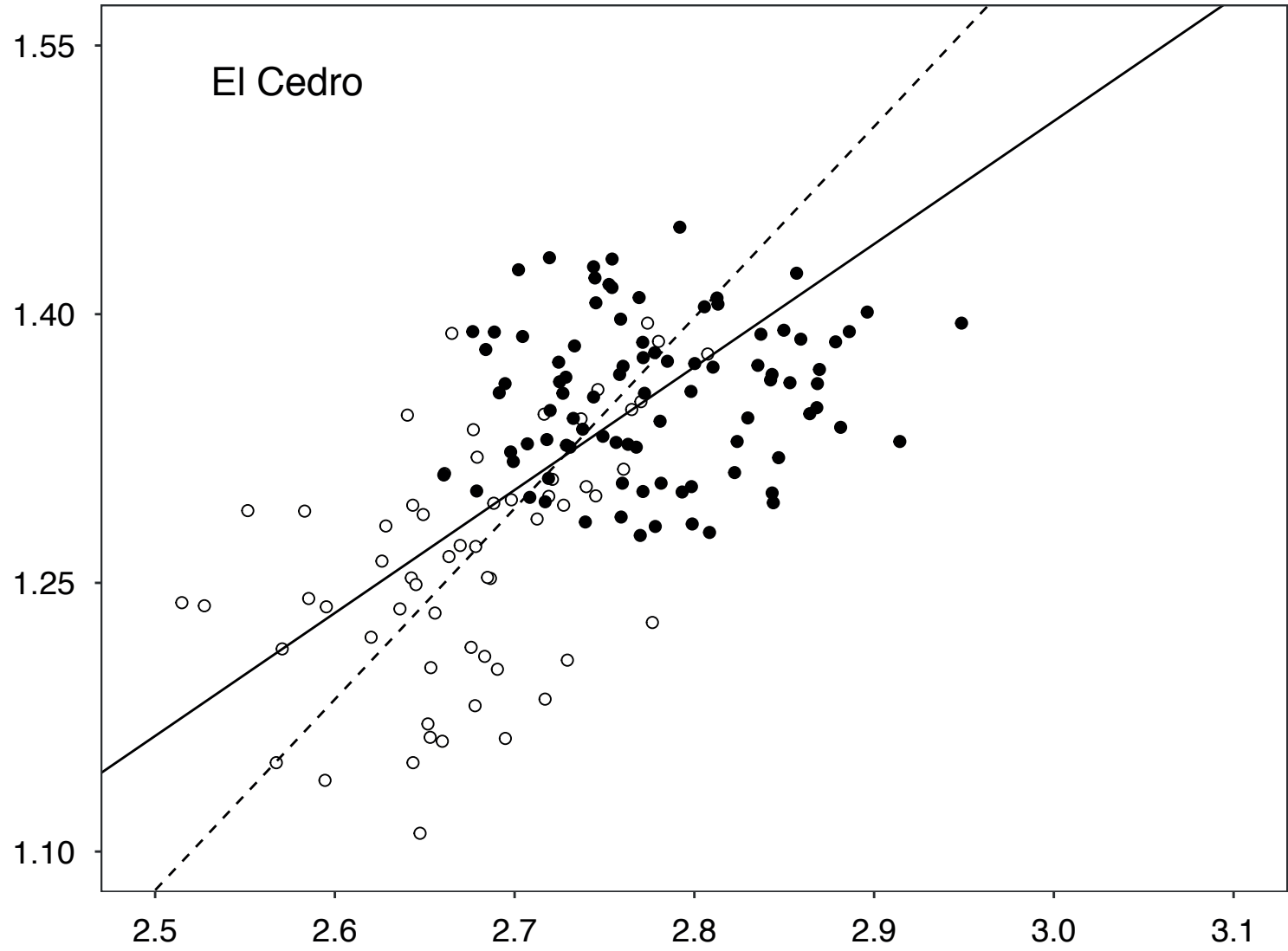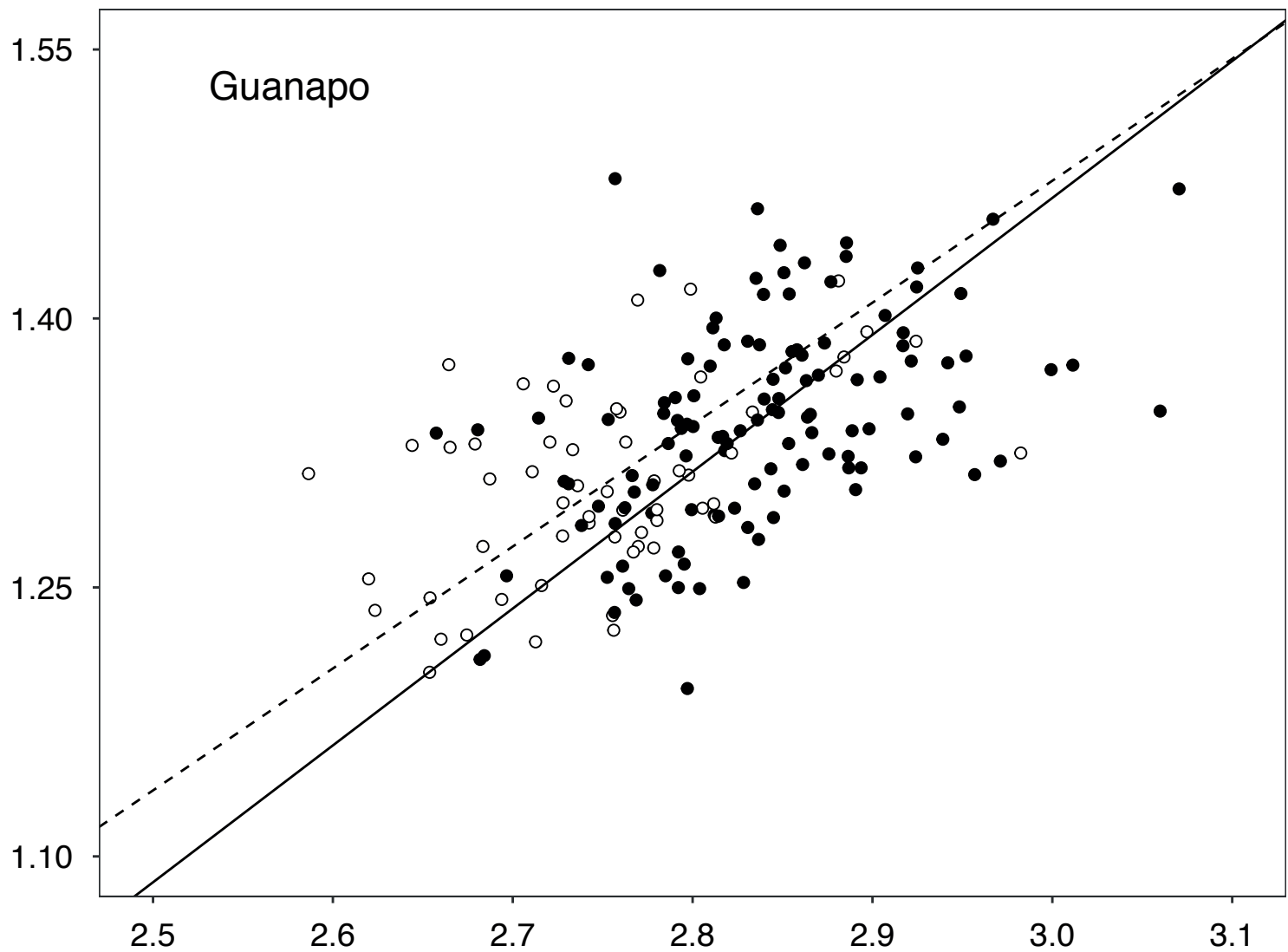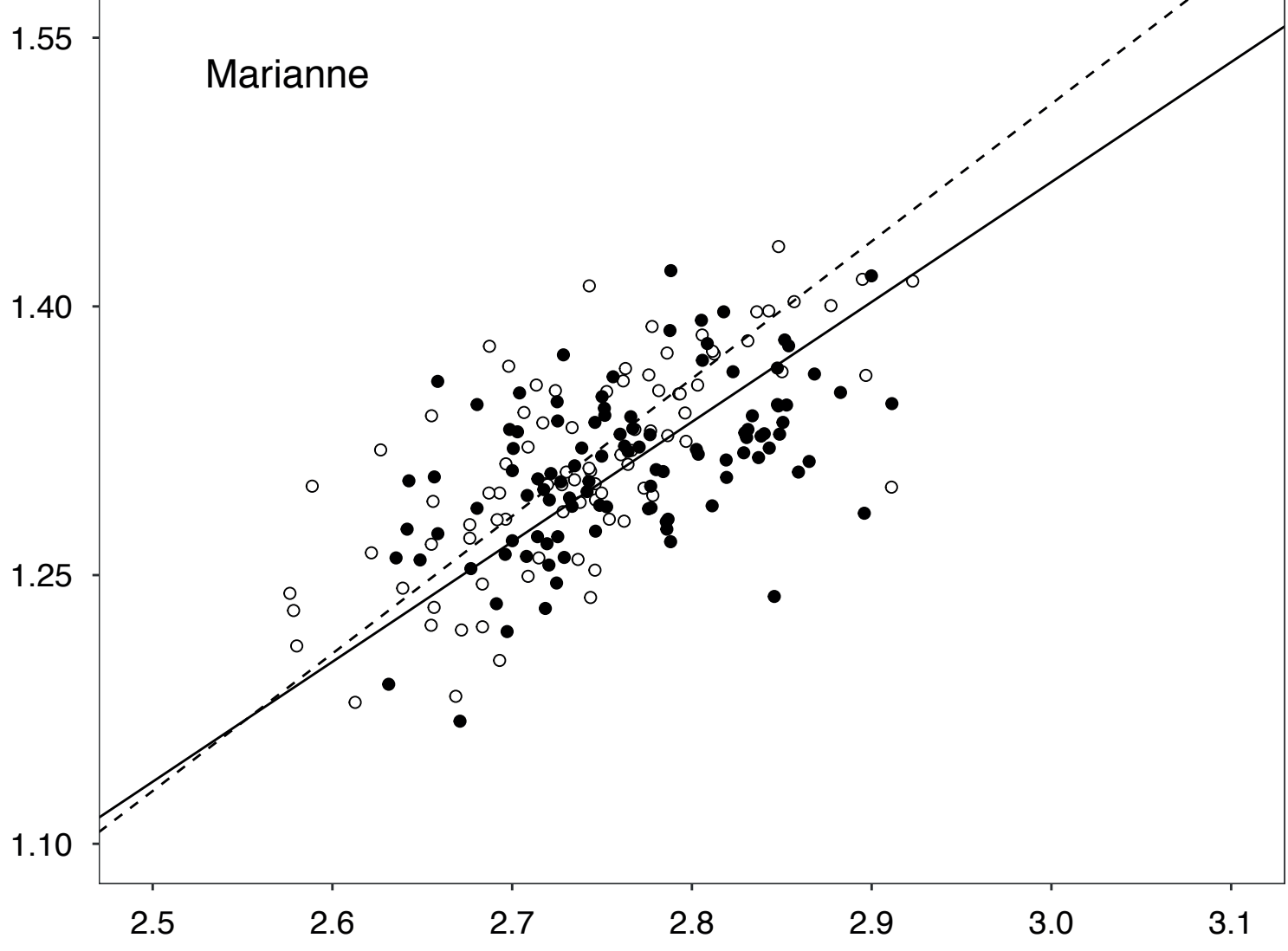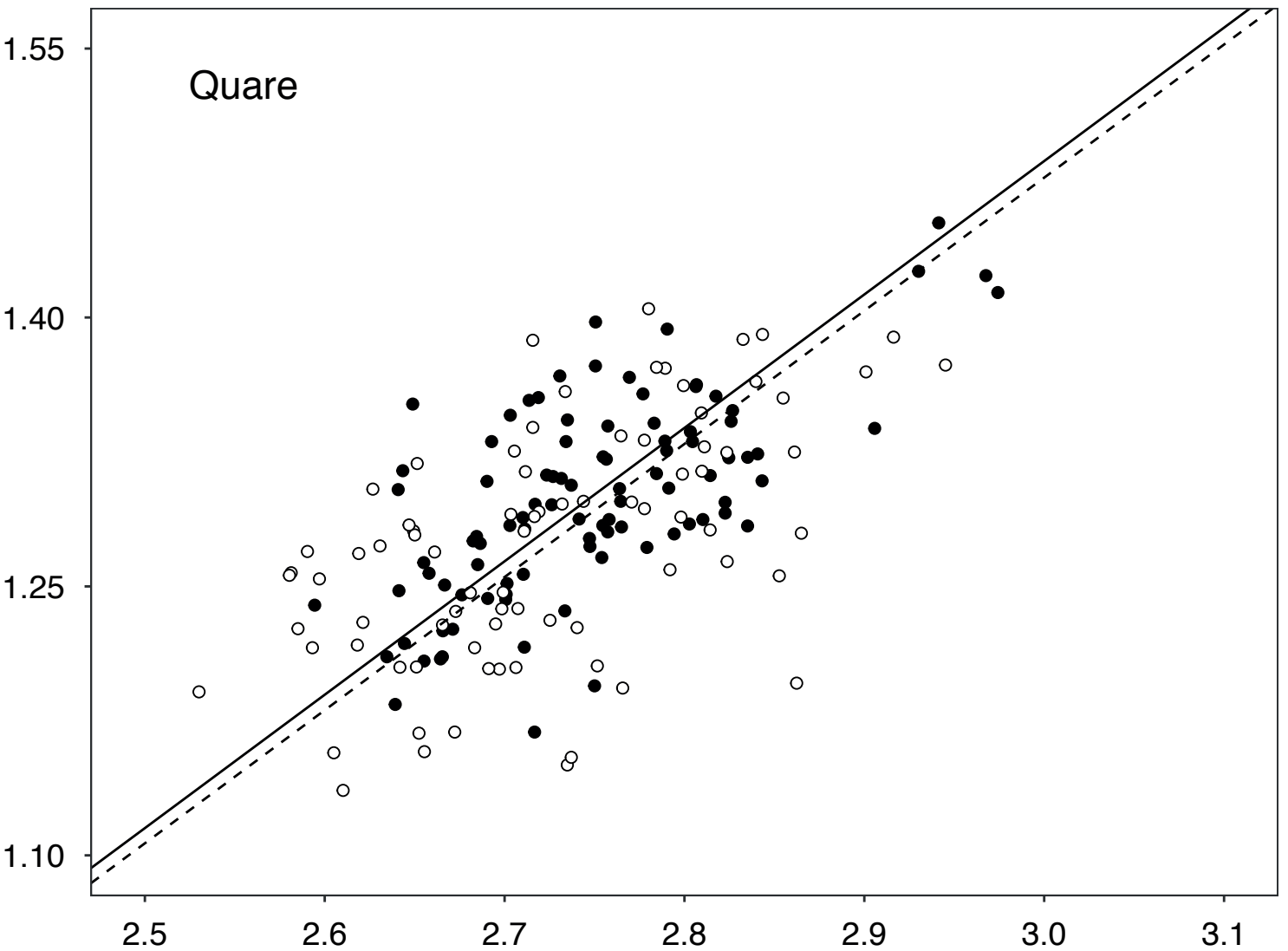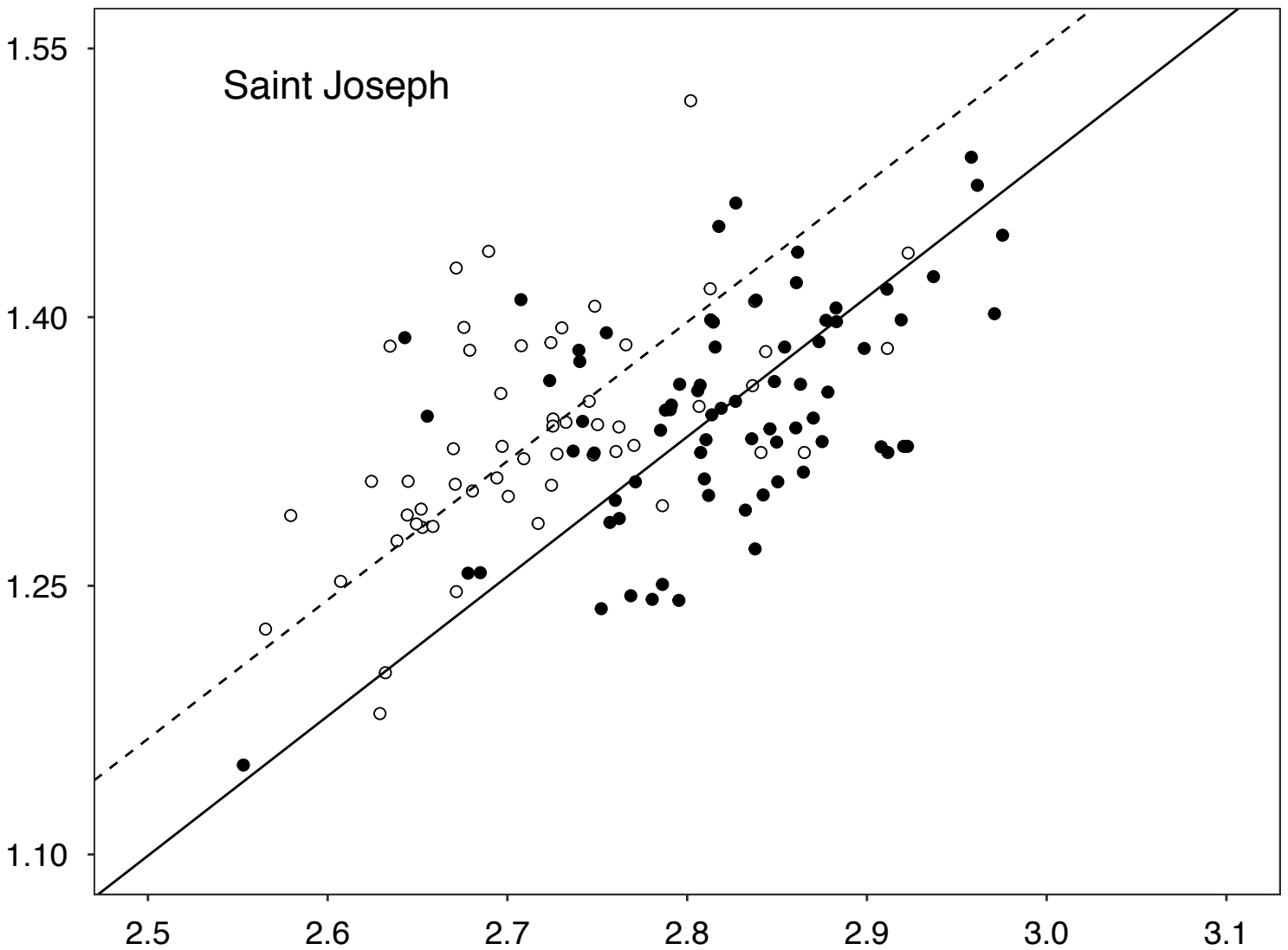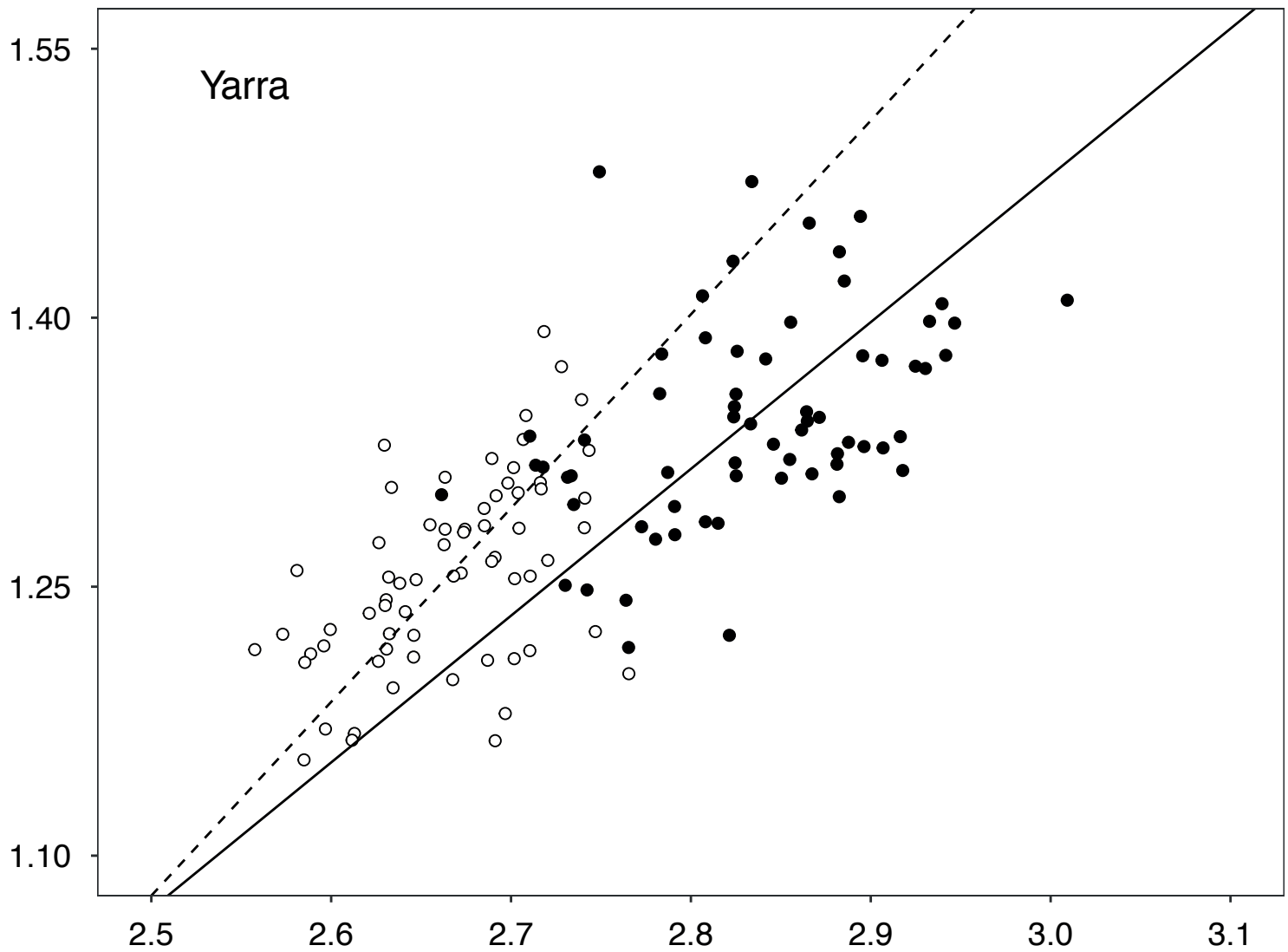

log Body length (mm)

○ High predation    ● Low predation

Supplement: Supplementary file 2 — Fig S1 [file ECE3-11-4564-s002.pdf]

log Gonopodium length (mm)

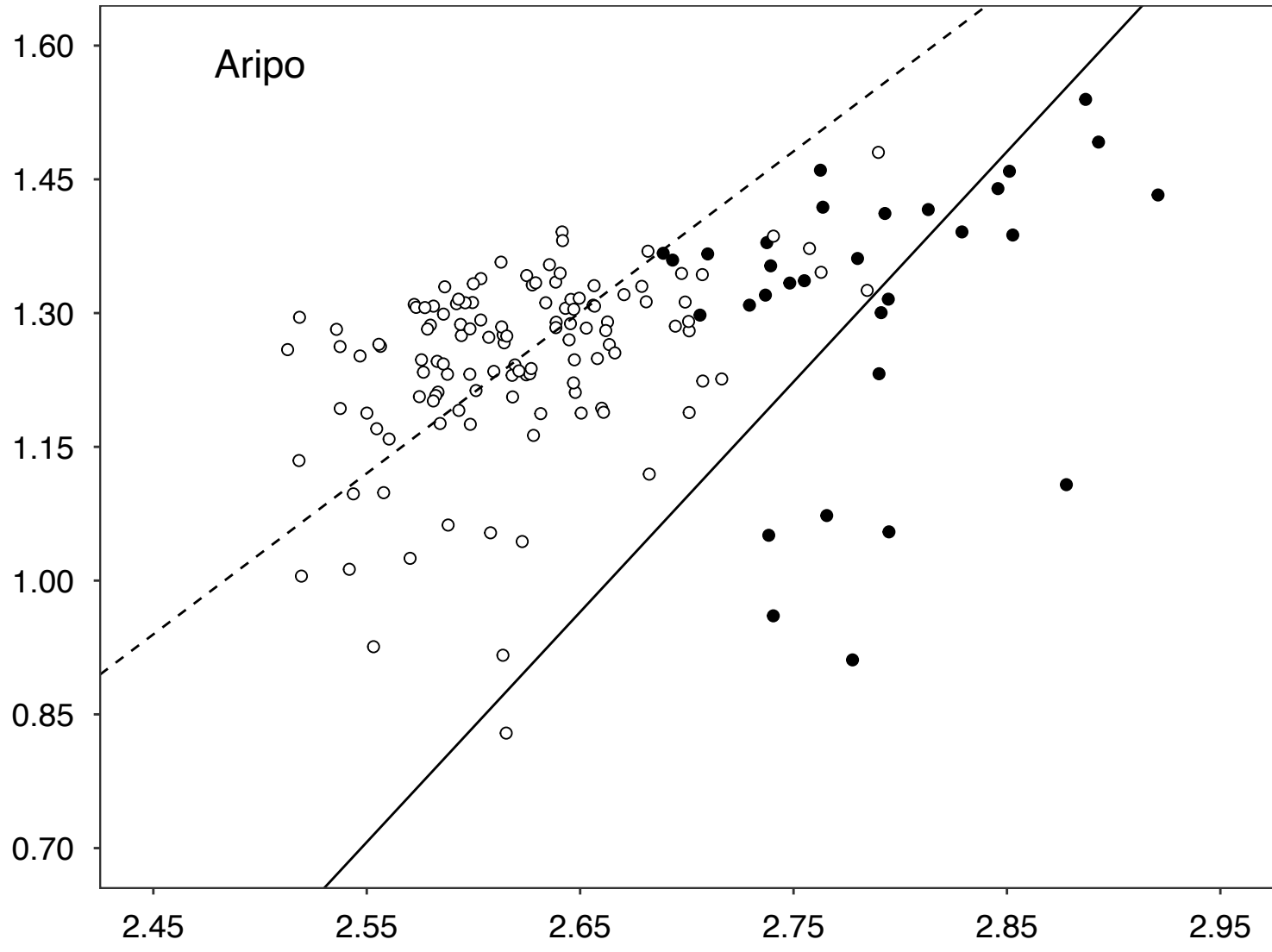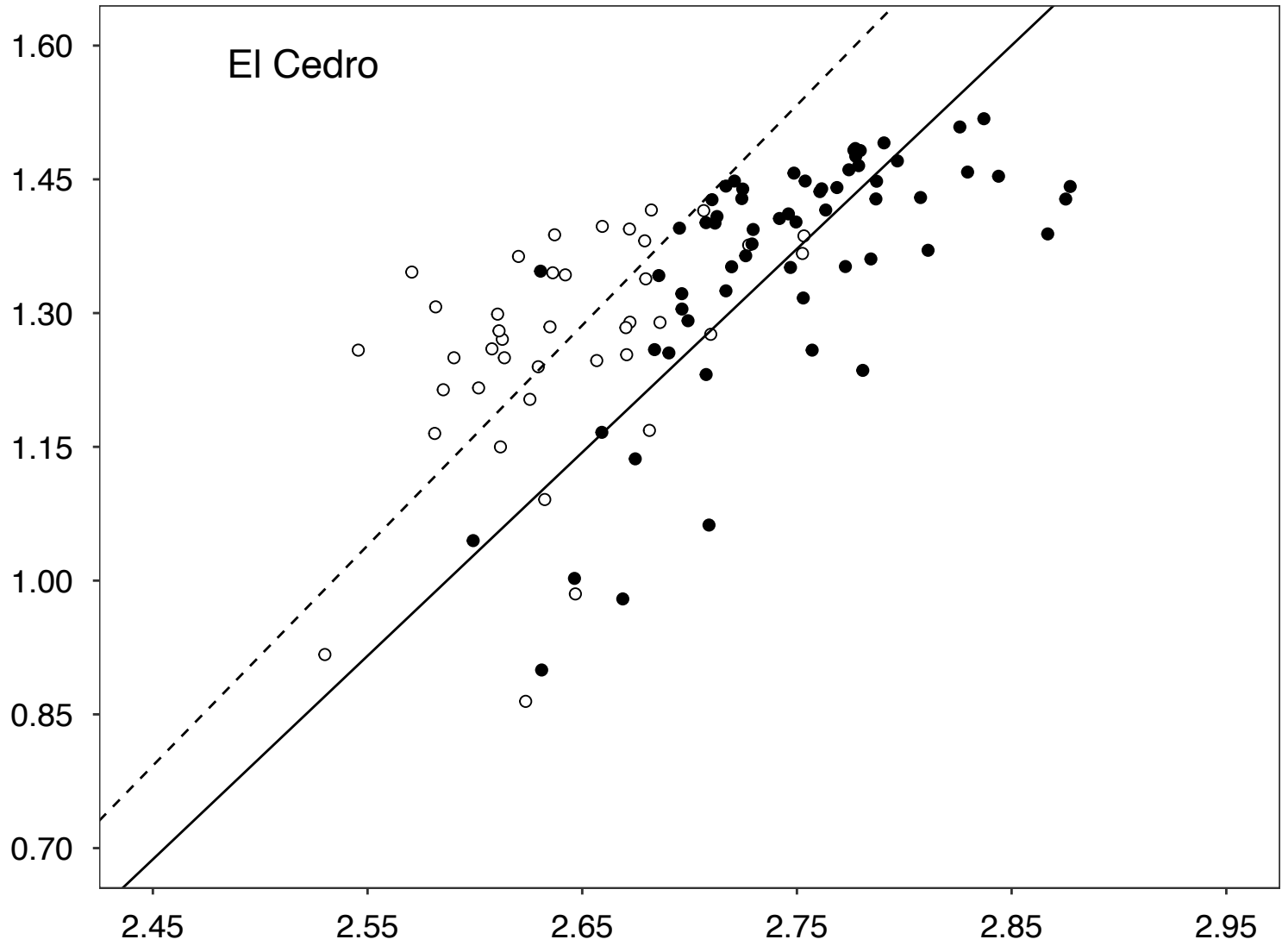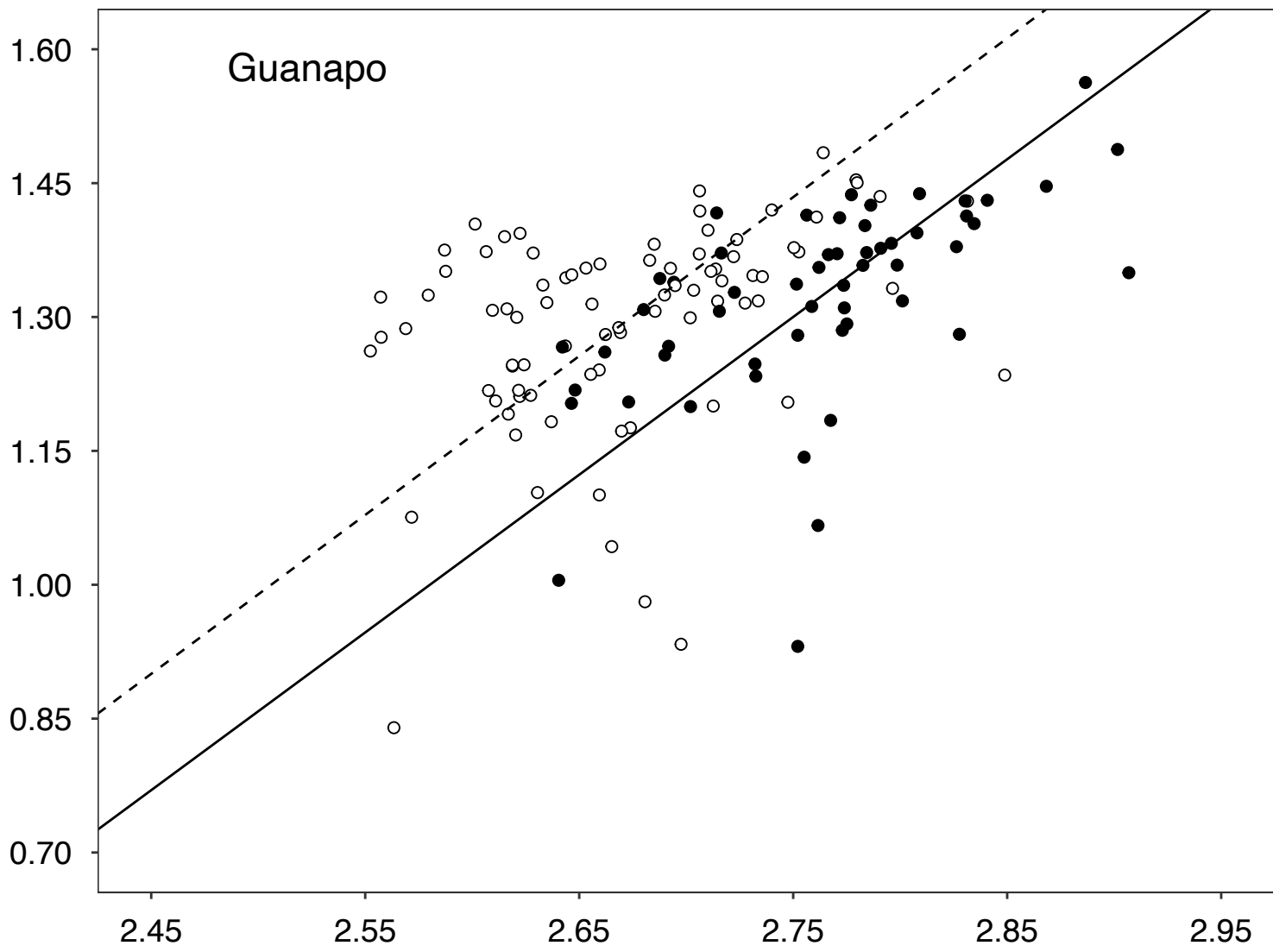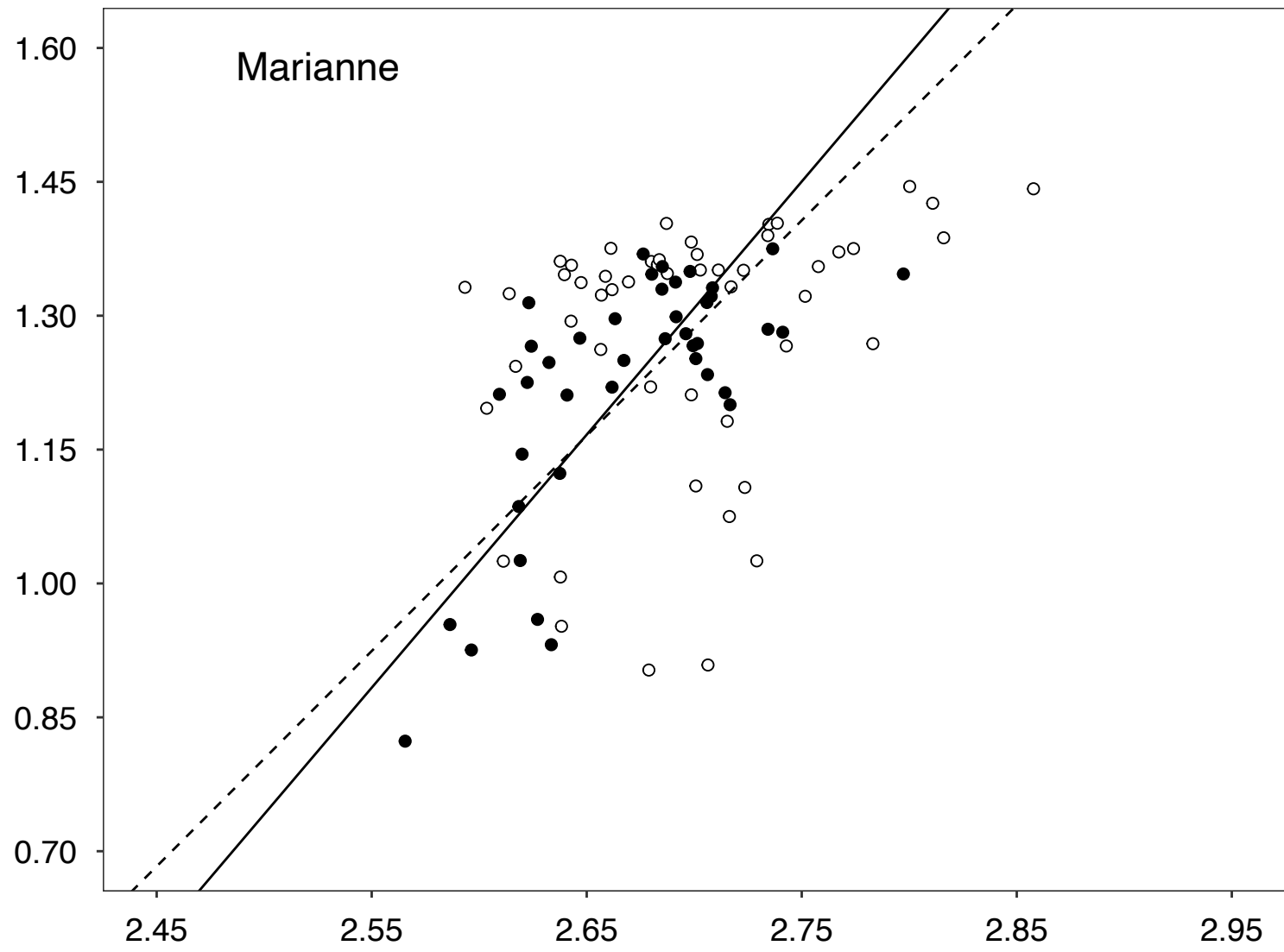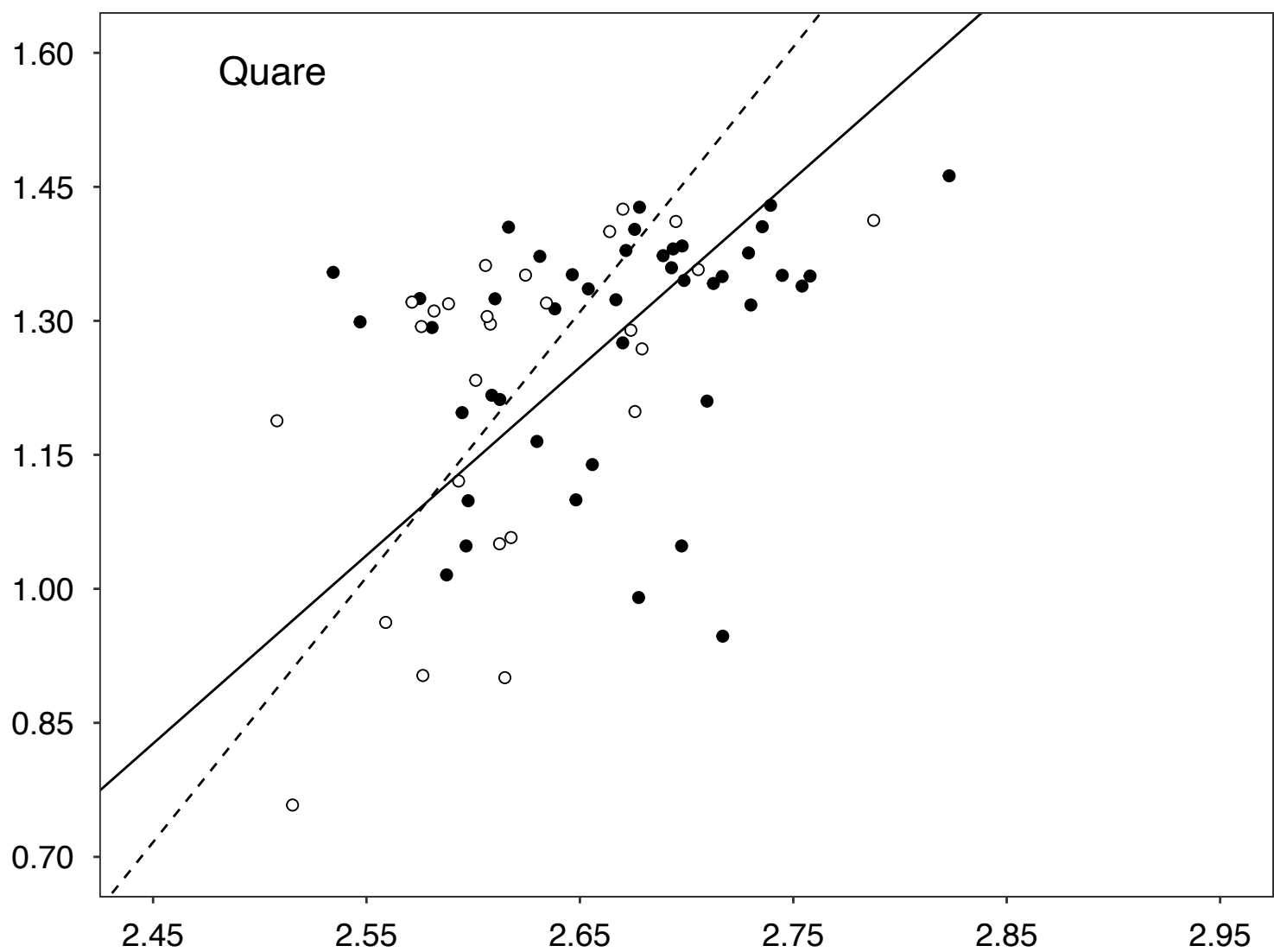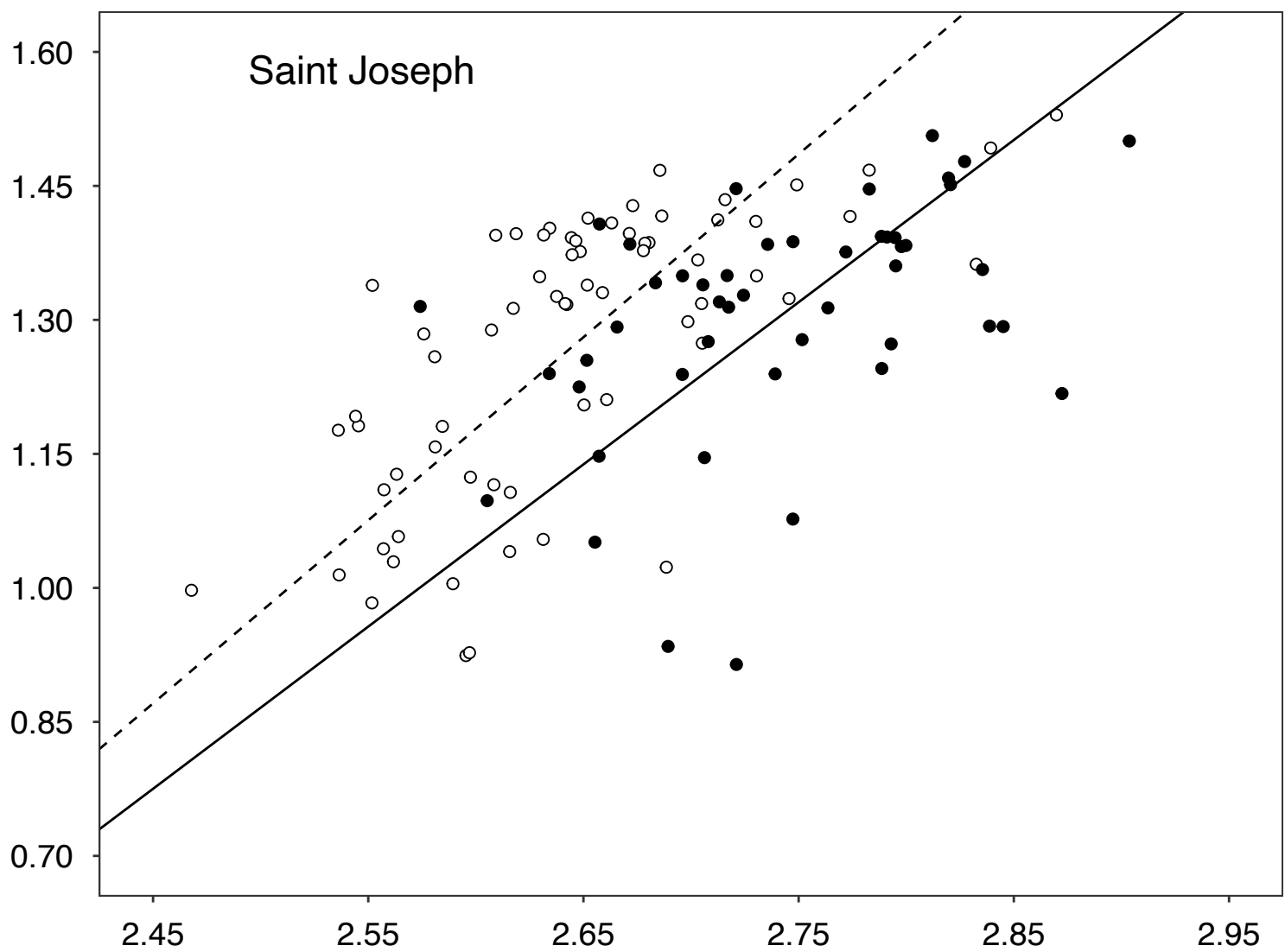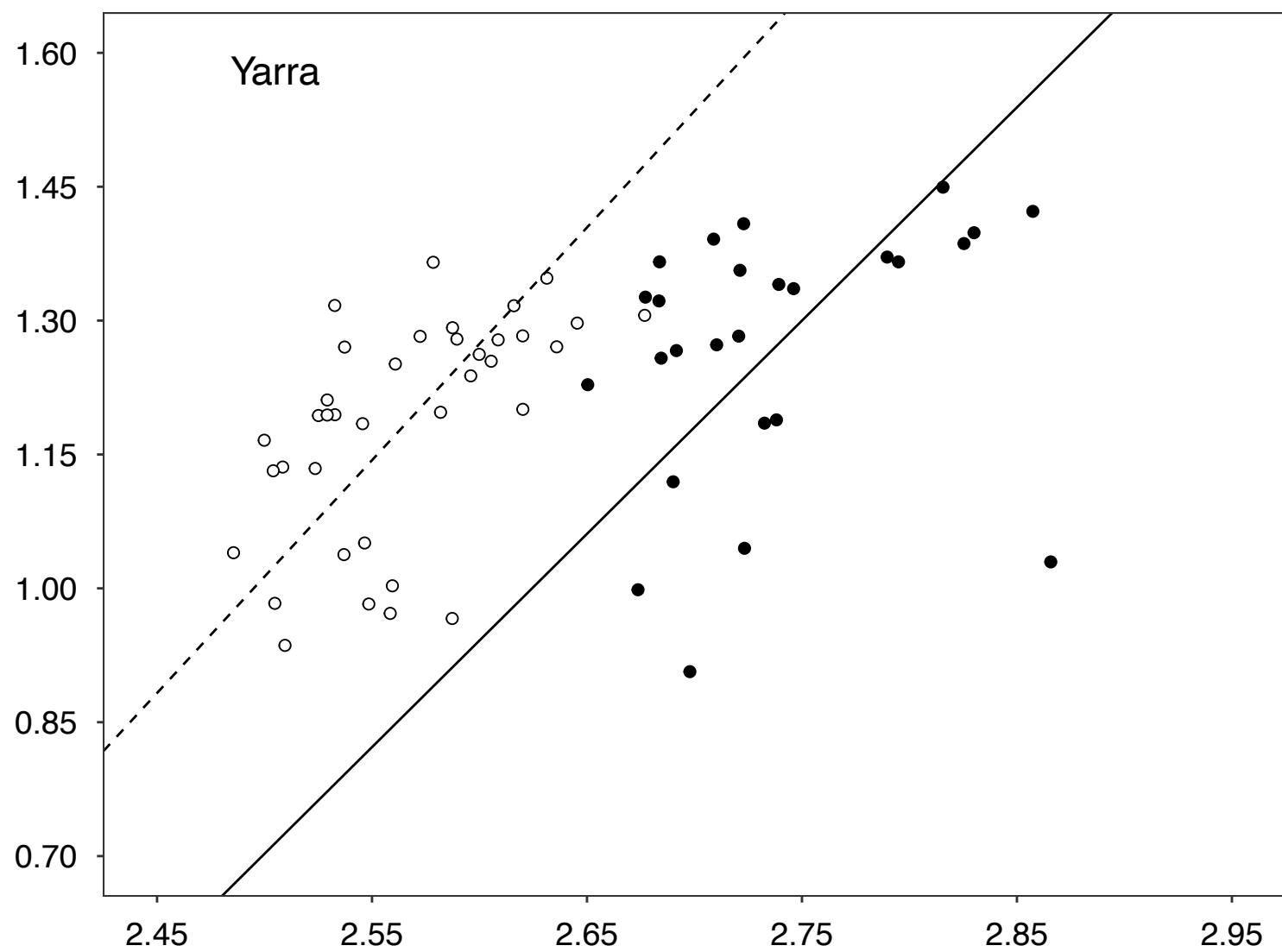

log Body length (mm)

○ High predation    ● Low predation

Supplement: Supplementary file 3 — Fig S2 [file ECE3-11-4564-s003.pdf]
